# Digitalization as a Driver of Innovation in Longterm-Care: Experiences From the German TCALL Project

**DOI:** 10.1093/geroni/igaf122.325

**Published:** 2025-12-31

**Authors:** Karin Wolf-Ostermann, Emily Mena, Claudia Stolle-Wahl, Benedikt Preuss, Heinz Rothgang

**Affiliations:** University of Bremen, Bremen, Bremen, Germany; University of Bremen, Bremen, Bremen, Germany; City University of Applied Sciences Bremen, Bremen, Bremen, Germany; Research Center on Inequality and Social Policy, University of Bremen, Bremen, Bremen, Germany; Research Center on Inequality and Social Policy, University of Bremen, Bremen, Bremen, Germany

## Abstract

Ensuring high-quality care is emerging as a key challenge for the future in Germany, particularly in light of demographic change and a shrinking workforce. Improving working conditions in long-term care through digital innovations seems to be a promising approach to improve quality of care and quality of work. The Transfer Cluster of Academic Teaching Nursing Homes in long-term care in Germany (TCALL) enables the implementation of digital innovations in regular operations and offers a direct transfer from academia to healthcare practice and back from healthcare practice to research and education. TCALL thus constitutes a conceptual space for innovative transfer activities and simultaneously provides a permanent physical environment for recursive innovation development, testing and implementation. Such transfer and innovation structures as regular and obligatory processes are currently lacking in Germany. For the first time, TCALL offers structures that directly contribute to the rapid and process-oriented development of the state of the art through innovative insights and products. We present the concept of TCALL, which has been implemented so far in three nursing homes in Bremen, and discuss examples of the implementation of digital solutions (e.g., an AI-based management for the prevention and documentation of falls). We identify the facilitators and barriers to implementing digital technologies in terms of structural and process-related factors, explore the implications of innovation implementation for personnel and organizational development, and share lessons learned from participatory approaches.

